# Real-Time Evaluation of Thyroid Cytology Using New Digital Microscopy Allows for Sample Adequacy Assessment, Morphological Classification, and Supports Molecular Analysis

**DOI:** 10.3390/cancers15174215

**Published:** 2023-08-23

**Authors:** Martina Verri, Stefania Scarpino, Anda Mihaela Naciu, Gianluca Lopez, Gaia Tabacco, Chiara Taffon, Emanuela Pilozzi, Andrea Palermo, Anna Crescenzi

**Affiliations:** 1 Unit of Endocrine Organs and Neuromuscular Pathology, Fondazione Policlinico Universitario Campus Bio-Medico, 00128 Rome, Italy; c.taffon@policlinicocampus.it (C.T.); a.crescenzi@policlinicocampus.it (A.C.); 2 Pathology Unit, Department of Clinical and Molecular Medicine, Sapienza University, Sant’Andrea University Hospital, 00189 Rome, Italy; stefania.scarpino@uniroma1.it (S.S.); gianluca.lopez10@gmail.com (G.L.); emanuela.pilozzi@uniroma1.it (E.P.); 3 Unit of Metabolic Bone and Thyroid Disorders, Fondazione Policlinico Universitario Campus Bio-Medico, 00128 Rome, Italy; a.naciu@policlinicocampus.it (A.M.N.); g.tabacco@policlinicocampus.it (G.T.); a.palermo@unicampus.it (A.P.)

**Keywords:** confocal laser microscopy, thyroid nodules, fine needle aspiration, Instant Digital Pathology, rapid on-site evaluation

## Abstract

**Simple Summary:**

The detection of thyroid nodules is increasing worldwide. Fine needle aspiration biopsy (FNA) is used to differentiate benign and malignant nodules and to address patient management; about 30% of patients receive indeterminate or inadequate cytological report. This clinical problem needs an innovative approach, overcoming the limits of traditional cytological diagnostics. Fluorescence laser confocal microscopes (FCM) is a new optical technique for allowing immediate digital imaging of fresh unfixed tissues, and it recently obtained attention for real-time assessment of sample adequacy and diagnostic evaluation for small biopsies and cytological samples. Herein we tested FCM technology for evaluating FNA thyroid samples at the procedure time and to assess the concordance between FCM evaluations, paired conventional cytology, and final histology of removed thyroid gland. We also analyzed the integrity of nucleic acids after FCM evaluation to assure viability for molecular assessment. Our results demonstrated that FCM might improve timely and accurate patient management.

**Abstract:**

Thyroid cytological examination, a key tool in preoperative thyroid nodule evaluation, is specific and accurate; some drawbacks are due to inadequate or indeterminate cytological reports and there is a need for an innovative approach overcoming the limits of traditional cytological diagnostics. Fluorescence laser confocal microscopes (FCM) is a new optical technique for allowing immediate digital imaging of fresh unfixed tissues and real-time assessment of sample adequacy and diagnostic evaluation for small biopsies and cytological samples. Currently, there are no data about the use of FCMs in the field of thyroid nodular pathology. The aims of this study were to test FCM technology for evaluating the adequacy of FNA samples at the time of the procedure and to assess the level of concordance between FCM cytological evaluations, paired conventional cytology, and final surgical histology. The secondary aim was to define the integrity of nucleic acids after FCM evaluation through NGS molecular analysis. Sample adequacy was correctly stated. Comparing FCM evaluation with the final histology, all cases resulting in malignant or suspicious for malignancy at FCM, were confirmed to be carcinomas (PPV 100%). In conclusion, we describe a successful application of FCM in thyroid preoperative cytological evaluation, with advantages in immediate adequacy assessment and diagnostic information, while preserving cellular specimens for permanent morphology and molecular analysis, thus improving timely and accurate patient management.

## 1. Introduction

The detection of nodular thyroid disease is increasing worldwide; ultrasound examination and fine needle aspiration biopsy (FNA) are considered the core tools to separate benign and malignant lesions and address patient management [1]. Thyroid cytological examination is specific and accurate; however, some drawbacks are due to inadequate or indeterminate cytological reports that involve about one third of the reports [2]. These non-informative results cause a clinical problem and there is a need for an innovative approach overcoming the limits of traditional cytological diagnostics. Optical digital technologies may represent a possible way to improve the diagnostic workflow. In recent years, Whole slide imaging (WSI) applications to histology have been characterized by a rapid development supported by the technological improvement of the hardware solutions for the scanning of the slides. WSI in cytology is more challenging and less used since it requires a different technical approach, increased scanning time, the multi z-stack levels scanning to focus all the cells leading to a consequent increase in images storage costs [3]. Several aspects have slowed down the progress of digital pathology, in particular digital techniques applied to histology and cytology still require the preparation of the conventional glass slide to be submitted for digital transformation using the slide scanner, resulting in a prolonged workflow. Recently, new optical imaging techniques are emerging for allowing immediate digital imaging of fresh unfixed tissues. Ex vivo fluorescent laser confocal microscopes (FCM) are able to generate digital microscopic images similar to H&E-stained tissue sections, from native biologic specimens, without any slide preparation. This kind of technology is known as Instant Digital Pathology [4]. FCMs use a laser source to obtain microscopic digital images through photons’ interaction with labelled and unlabeled tissue components. Some drops of acridine orange are commonly used to label nucleic acids and enhance nuclear evaluation. The FCMs are mainly applied to accelerate intraoperative evaluation of resection margins in skin and urological surgery [5,6,7]. More recently, new protocols have been optimized for immediate assessment of small tissue specimens, such as image-guided core needle biopsy and endoscopic biopsy specimens [8,9,10]. The obtained digital images allow for rapid assessment of sample adequacy, real-time diagnostic opinion from remote hospitals, and digital sharing among pathologists. FCM morphological evaluation of cells and tissue does not cause any damage to the specimen, which remains unaltered during the digital imaging. The process takes about 1 min. After FCM imaging, the fresh sample is recovered from the device and it may be formalin fixed and paraffin embedded (FFPE) for permanent histology and ancillary techniques. Recent studies and our previous results on pancreatic endoscopic ultrasound-guided fine needle biopsy from solid pancreatic lesions [11,12,13] strongly support the utility of this technique in the diagnostics of small biopsy and cytological samples. Currently, there are no data about the possibility of using FCMs in the field of thyroid nodular pathology. Most important, there are no established protocols for such type of samples. The aims of this project were (I) to test the performance of the FCM Vivascope 2500 (Vivascope, Munchen, Germany) on thyroid cytology specimens for evaluating the adequacy of the sample at the time of the FNA procedure and (II) to assess the level of concordance between FCM cytological evaluation, paired conventional cytological diagnosis, and final histology of the surgically removed thyroid gland. The secondary endpoint is to define the integrity of nucleic acids in the FNA material after FCM evaluation by assessing the feasibility of molecular analysis on the FNA cellblock of post-Vivascope samples and its concordance with molecular analysis performed on the paired surgical tissue.

## 2. Material and Methods

### 2.1. Study Design

This prospective, blinded, and single-center study took place at the Unit of Endocrine Organs and Neuromuscular Pathology of Fondazione Policlinico Universitario Campus Bio-Medico, Rome, Italy. Patients’ enrollment was conducted at the thyroid outpatient clinic of the Metabolic Bone and Thyroid Disorders Unit of the Fondazione Policlinico Universitario Campus Bio-Medico between April 2020 and September 2021. Molecular analysis was performed at the Pathology Unit of S. Andrea University Hospital, Rome. The study protocol adhered to the Declaration of Helsinki and to the International Conference on Harmonization Good Clinical Practice, receiving approval of local ethics committees (31/19 PAR ComEt CBM from 26 July 2019). All participants granted informed consent.

Patients’ eligibility criteria were (1) ≥18 years old; (2) at least one thyroid nodule with medium-high ultrasound risk of malignancy (Thyroid Imaging Reporting & Data System [TI-RADS] score ≥ 3) [14]; (3) referral to our outpatient clinic for thyroid FNA, according to clinical guidelines [15]; and (4) signature of informed consent to participate in this study. Once thyroid FNA cytology was reported, only those subjects with at least one nodule categorized as indeterminate high risk, suspicious, or malignant and warranting thyroid surgery were included in the study. Twenty patients were finally enrolled in this study.

### 2.2. Sample Preparation

**Figure 1:**
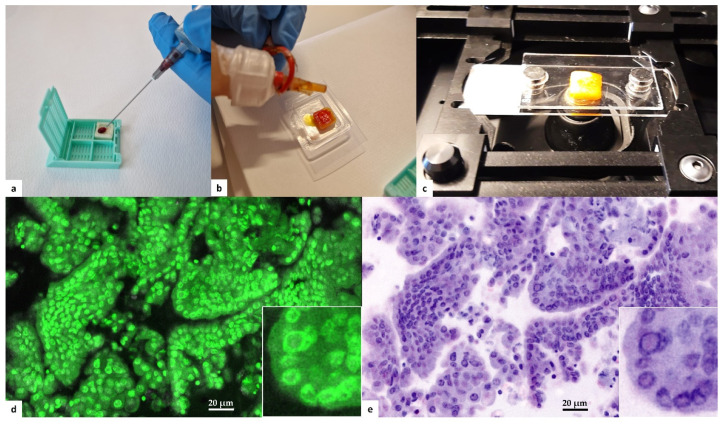
Protocol for instant digital imaging. (**a**) Cytological specimen is loaded of the Cytomatrix immedi-ately after FNA sampling of the thyroid nodule. (**b**) The specimen is promptly dropped with acridine orange solution directly on the holder. (**c**) After washing with saline, the Cytomatrix is placed be-tween two dedicated glass slides for Vivascope imaging. (**d**) Digital imaging uses reflectance (785 nm) and fluorescence (488 nm) modalities: acridine appears green florescence in the nuclei. A nu-clear pseudoinclusion is recognizable in the high-power field inset. (**e**) Proprietary software makes the conversion in H&E pseudocolor. The nuclear pseudoinclusion is evident in the high-power field inset.

Fine needle aspiration (FNA) of thyroid nodules was performed using a 22-G needle under US guidance. Two passes were obtained for each nodule. The first pass was smeared on a conventional glass slide, fixed with alcohol based cytospray (Bio-fix, Bio-Optica, Milan, Italy), and submitted for cytological evaluation. Cellular material from the second pass was delivered on a commercial polymeric scaffold for cytological samples (Cytomatrix, UCS Diagnostics, Rome, Italy). Cytomatrix is a CE-IVD diagnostic tool intended to produce a cellblock directly from the needle, avoiding needle washing and the following laboratory preparations. Cytomatrix consists of a naturally derived, foam-like porous substrate endowed with a net positive surface charge that helps retain the cytological and microhistological material obtained by FNA/FNB sampling. After loading cells on the scaffold (Figure 1a), it was immediately prepared for Vivascope analysis. The scaffolds were dropped with acridine orange solution (0.6 mM; Sigma-Aldrich, St. Louis, MO, USA) (Figure 1b) for 20 s before being rinsed with physiological saline solution to wash off any excess of acridine solution. The samples were quickly drained on absorbent paper, and then placed between two dedicated glass slides (Figure 1c). Glass sandwiches were then positioned in the FCM stage for image acquisition and conversion in H&E pseudocolor (Figure 1d,e). FCM images were stored on a dedicated hard disk, according to a patient identification number and intervention date. After confocal analysis on fresh cellular specimens, samples were formalin-fixed and paraffin-embedded for permanent microscopic diagnosis along with paired routine FNA samples. Both FCM diagnosis and conventional cytological diagnosis were reported in agreement with Italian and Bethesda systems for reporting thyroid cytology [16,17]. Since Vivascope imaging with fluorescent dye does not allow the assessment of slight chromatin alteration, we decided to put in one single category (Follicular lesion) all indeterminate cytological samples. Two expert pathologists in the field of thyroid cytology observed in blind all digital images, conventional FNA cytological smears, and permanent Cytomatrix FFPE. Patients with clinical indication for thyroid surgery were definitely enrolled for this study. Removed thyroid glands were formalin-fixed and paraffin-embedded for routine histological examination. Diagnoses were reported in agreement with WHO Classification of Tumors of Endocrine Organs 2017 [18]. Seriate paraffin sections from both Cytomatrix and surgically removed glands were submitted for molecular analysis at the Pathology Unit of S. Andrea University Hospital of Rome, to investigate the complete preservation of biomolecular characteristics of the cells after the Vivascope procedure and to confirm the availability of these specimens for genomic testing.

### 2.3. Instant Digital Microscopy Instrument

The FCM Vivascope 2500M-G4 (Vivascope GmbH, Munich, Germany; Caliber I.D.; Rochester, NY, USA) combines two different lasers that enable tissue examination according to reflectance (785 nm) and fluorescence (488 nm) modalities. Magnification reaches × 550 and the reconstructed image is a collection of mosaic images with a maximum total scan area of 25 × 25 mm. The microscope is equipped with a water immersion objective with 38× magnification and a numerical aperture of 0.85. Laser illumination of the specimen allows for the building of the microscopic image. The short-wave laser demonstrates the cell nuclei marked with fluorescent acridine orange dye before the examination. The cytoplasmic and extracellular structures, on the other hand, are shown by the reflected light of the long-wave laser. A built-in algorithm transforms the recorded values into an image similar to haematoxylin/eosin (H&E) staining, in which the nuclei are shown in blue/violet, whereas cytoplasm and extracellular structures are transformed in pink [19]. Balance of the staining intensity of nuclear and cellular/extracellular structures is modified by a pathologist regulating the intensity of the illuminating lasers.

### 2.4. Molecular Analysis

A permanent paraffin section of Cytomatrix recovered after FCM evaluation and a paraffin section of paired lesion in surgically removed thyroid were tested for hot spot mutation. Cases that resulted in wildtype for hot spot mutation were submitted for the assessment of gene rearrangements. Next Generation Sequencing (NGS) analysis was performed using the Oncomine Focus panel assay on the Ion Torrent platform. Three paraffin sections of 5-micron thickness were cut from the representative block of each surgical case and 6 paraffin sections 5-micron thick were cut from the corresponding Cytomatrix-embedded cellblock. One section for each case was stained with hematoxylin/eosin and assessed for quality (tumor fraction ≥ 50%) by an expert pathologist. A QIAmp DNA FFPE Tissue Kit (Qiagen, Hilden, Germany) was used to isolate DNA following the manufacturer’s instructions. A High Pure MiRNA isolation Kit (Roche, Basilea, Switzerland) was used to purify total RNA and prepare samples enriched for small RNAs (<100 nucleotides) according to the manufacturer’s instructions. Nucleic acid concentrations were determined by a Qubit 2.0 Fluorimeter using fluorescence-based quantification assays, respectively, for DNA and RNA (Qiagen, Hilden, Germany). Complementary DNA (cDNA) synthesis panel was carried out using SuperScript™ VILO™ cDNA Synthesis Kit (Thermo Fisher Scientific, Waltham, MA, USA).

Library preparation was carried out using the Oncomine Assay™ comprising the DNA Oncomine™ Focus Assay (Thermo Fisher Scientific) and RNA Oncomine™ Fusions assay (Thermo Fisher Scientific) following the manufacturer’s instructions using a total of 10 ng input DNA and/or RNA per sample. The DNA panel can identify hotspot mutations in the following genes: *AKT1*, *ALK*, *AR*, *BRAF*, *CDK4*, *CTNNB1*, *DDR2*, *EGFR*, *ERBB2*, *ERBB3*, *ERBB4*, *ESR1*, *FGFR2*, *FGFR3*, *GNA11*, *GNAQ*, *HRAS*, *IDH1*, *IDH2*, *JAK1*, *JAK2*, *JAK3*, *KIT*, *KRAS*, *MAP2K1*, *MAP2K2*, *MET*, *MTOR*, *NRAS*, *PDGFRA*, *PIK3CA*, *RAF1*, *RET*, *ROS1*, and *SMO*. The RNA panel can identify rearrangements in the following genes: *ALK*, *RET*, *ROS1*, *NTRK1*, *NTRK2*, *NTRK3*, *FGFR1*, *FGFR2*, *FGFR3*, *MET*, *BRAF*, *RAF1*, *ERG*, *ETV1*, *ETV4*, *ETV5*, *ABL1*, *AKT3*, *AXL*, *EGFR*, *ERBB2*, *PDGFRA*, and *PPARG*.

Nineteen copy number variant (CNV) targets are also included in the Oncomine™ Focus Panel. Template preparation was performed on the Ion Chef System (Thermo Fisher Scientific) using the Ion PGM Hi-Q Chef Kit and/or the Ion One Touch™ 2 System using the Ion PGM Template OT2 200 Kit. Sequencing was performed using the Ion PGM Hi-Q Sequencing Kit on the Ion Torrent Personal Genome Machine (Ion PGM). 

Analysis was carried out using Ion Torrent Suite™ Browser version 5.0 and Ion Reporter™ version 5.0. Variants were identified by Ion Reporter filter chain 5% Oncomine™ Variants (5.0). A cut off of 500× coverage was applied to all analyses. 

### 2.5. Fluorescence In Situ Hybridization (FISH) Analysis

FISH analysis was performed on RET- cases showing RET fusion at the NGS analysis. A commercially available dual color break-apart RET probe (Empire Genomics) was used following the manufacturer’s instructions. Briefly, for FISH on FFPE tissue sections and Cytomatrix sections, thin sections (5–6 μm) were treated with a pretreatment Poseidon Kit following the manufacturer’s instructions (Resnova, Rome, Italy). Sections were viewed under a NIKON fluorescent microscope with appropriate filters (NIKON Instrument, Florence, Italy).

## 3. Results

The study population included 20 patients with neoplasms of the thyroid gland: 6 male and 14 female, of age ranging from 30 to 82 years. The diameter of the nodules ranged from 7 to 70 mm (20.35 ± 15.84 mm, mean ± SD) and the US risk ranged from 3 to 5 in agreement with the TIRADS system. Serum TSH ranged from 0.56 to 3.2. 

All the patients received a cytological report, according both to the Italian Reporting System for Thyroid Cytology and The Bethesda System for Reporting Thyroid Cytopathology classification.

Among the 20 samples, two (10%) were classified as TIR3A (AUS/FLUS), seven (35%) as TIR3B (FN/SFN), four (20%) as TIR4 (SM), and seven (35%) as TIR5 (Malignant). 

The Vivascope analysis was obtained successfully in all 20 cases. The digital images of the thyroid cells loaded on Cytomatrix holder were collected in the database. 

**Figure 2:**
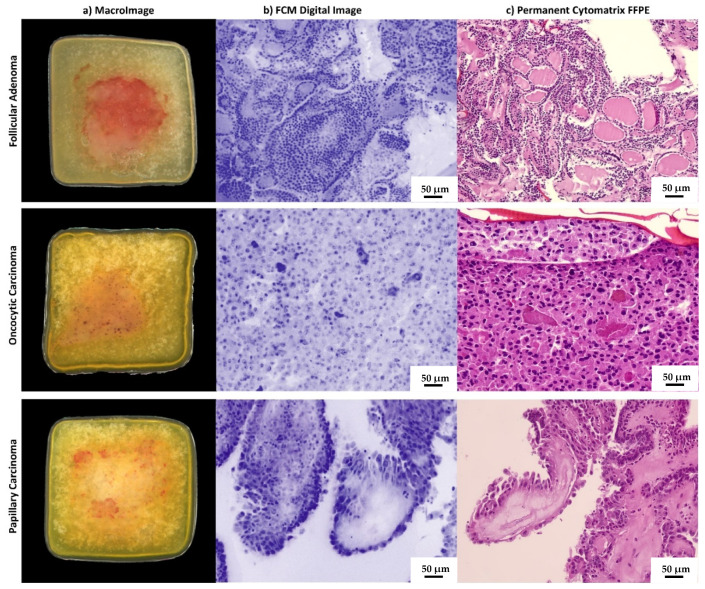
Imaging of cases from the study. Macro-images of the holder with FNA sample as it is placed within the microscope slot are shown in the column (**a**). Vivascope digital immediate images converted in pseudocolor are shown in column (**b**). Conventional H&E-stained sections from FFPE Cytomatrix blocks are shown in column (**c**). The case from the first line is a follicular adenoma in agreement with histological diagnosis on the surgically removed thyroid gland. The case in line 2 is an oncocytic cell carcinoma, and in line 3 there is a papillary carcinoma, hobnail subtype.

For all 20 samples loaded in Cytomatrix holder, Vivascope FCM allowed having a macro-image and the digital image of the fresh cellular material collected during the FNA procedure (Figure 2a,b). Vivascope allowed satisfactory digital imaging and all 20 samples were defined as adequate for diagnostic purposes at the FCM evaluation. 

The morphological characteristics evaluated in the digital images were architectural atypia, nuclear irregularities, and cytoplasmic features. Based on these features, Vivascope digital images were classified as Follicular lesion (i.e., indeterminate categories of reporting systems), Suspicious for Malignancy, and Malignant by two pathologists skilled in thyroid cytology. In detail, among 20 samples, seven (35%) were defined as follicular lesion, five (25%) as suspicious for malignancy, and eight (40%) as Malignant. 

All 20 samples evaluated on the permanent Cytomatrix FFPE were defined as adequate for diagnosis. Their final microscopic examination confirmed the paired VivaScope assessment (Figure 2c). Overall, there was complete diagnostic agreement between the two pathologists. When comparing the FCM evaluation with the final histology on the surgically removed thyroid glands, all cases reported at FCM as malignant or suspicious for malignancy were confirmed to be carcinomas (PPV 100%). Among seven cases classified as follicular lesions at FCM, four resulted in follicular adenomas and three resulted in carcinomas at histology (two oncocytic Hurthle cell carcinomas and one follicular subtype of papillary carcinoma).

The patients’ features and the comparison of ex vivo confocal laser microscopy, cytological diagnosis, and conventional histopathology are summarized in Table 1.

For molecular analysis, DNA and RNA extraction was completely successful in 18 out 20 cases. In one unsuccessful case, the DNA extraction failed on Cytomatrix FFPE and was effective on surgical tissue, while RNA extraction failed on both; in the second case, the nucleic acid isolation was unsuccessful on both Cytomatrix and surgical tissue. These two cases were excluded from the statistical evaluation. DNA isolated from Cytomatrix sections showed range of between 1.4 and 51.2 ng/µL, while DNA obtained from FFPE sections of surgical samples ranged between 27.1 and 393.4 ng/µL.

We identified oncogenic mutations in 9/18 (50%) samples, while 9/18 (50%) samples resulted in wildtype for the investigated mutations. Among mutated samples, NGS-based analyses revealed that 8/9 (90%) samples had a point mutation on the exon 15 of the BRAF gene (p.V600E, COSM476), while in only one sample (10%) we identified a point mutation on the exon 3 of the HRAS gene (p.Q61R, COSM499). Comparing the mutational analysis in Cytomatrix FFPE and paired surgical specimens, we observed a complete concordance of genetic mutational status, although the hot spot mutations sometimes showed a different allelic frequency.

**Figure 3:**
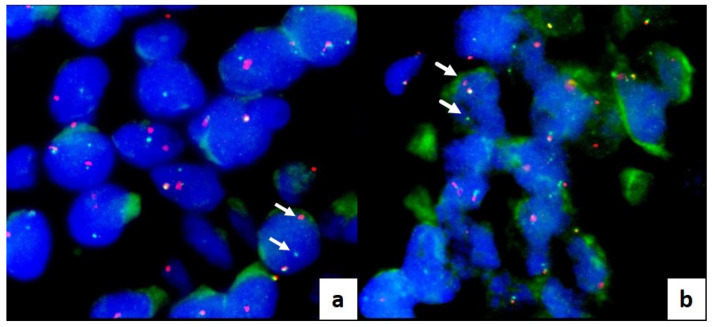
FLUORESCENT IN SITU HYBRIDATION (FISH) on interphase nuclei, labeled with DAPI (4′,6-diamidino-2-phenylindole): translocation analysis of the RET gene performed on FFPE Cytomatrix (**a**) and thyroid surgical tissue (**b**). Arrows indicate the presence of the translocation. The probe comes labeled in red and green. Probe: dual color break apart (Empire Genomics).

Fusions were investigated in cases resulting in wildtype for BRAF or other gene mutations. Among the nine wildtype cases, eight (90%) cases were negative for fusion investigation, and one (10%) revealed CUX1-RET (C10R12) gene fusion. This RET gene fusion was confirmed by break-apart RET FISH assay, which shows separate red and green signals in tumor cells both in Cytomatrix FFPE sections and final histological sections of surgical samples (Figure 3a,b).

All results of NGS analysis are shown in Table 2.

## 4. Discussion

The current prevalence of thyroid lesions in the general population varies from 2 to 65% depending on diagnostic techniques [20], and most of them are asymptomatic with normal thyroid hormone secretion [21]. This challenging clinical context requires new diagnostic technologies to help endocrinologists to better manage the patients. In this study, we demonstrated for the first time the feasibility of immediate cytological analysis of cellular samples from fine needle aspiration biopsy of thyroid nodules, using confocal laser-scanning microscopy. The diagnostic utility of FCM real-time assessment in thyroid cytology regards the availability of accurate information about sample adequacy at the time of FNA without smearing and staining of the cells. The specimen preservation during the morphological analysis is a key feature of FCM technology and a growing interest is focused on bedside evaluation of cytological and small biopsy samples [11,13,22]. The FCM protocol allows estimating the adequacy of the cellular population both for cells amount and for quality of morphological details on fresh material, avoiding manipulation of the cytological material. Moreover, the matrix used as holder for the collection of cells is directly formalin-fixed for permanent paraffin embedding after FCM analysis, overcoming handling variability and cell loss during cell-block preparation. The cellular inclusion obtained with this method contains the intact original FNA material and supports cytological diagnostics, and, when required, immunophenotyping and molecular analysis. Primarily, FCM application may be conceived in nodules with indeterminate cytological features. These in fact represent a growing clinical problem and molecular testing is proposed to improve the diagnostic accuracy of FNA and to reduce the need for diagnostic surgery [23]. To assure the adequacy of the sample, the rapid on site evaluation (ROSE) has been suggested [24]; however, the need for additional human resources limits the routine use of this approach. Moreover, the smeared cellular material required for ROSE may be sub-optimal for immunohistochemical panels or multigene molecular test. Ex vivo confocal laser-scanning microscopes avoid any damage in cells and tissue during the evaluation and, after the morphological analysis, the samples turn into a conventional cellblock as suggested for multi-target analysis [25,26]. Vivascope evaluation using confocal scanning of multiple optical plans consents a quantitative evidence of neoplastic cells amount in the specimens and their percentage among total cellularity. This information is of value, especially when the use of molecular or immunohistochemical testing is expected. Adequacy for molecular testing is fundamental in specific settings, such as anaplastic carcinoma or locally invasive non-operable tumors that require molecular assessment on the cytological sample for planning adequate target therapies [27]. In our study, two cases with less than 500 cells in the cytological sample were sufficient for morphological appraisal but were unable to give good quality nucleic acids amount. This event is a known limit for NGS analysis [28]. Based on this result, we may argue the utility to use such a cut-off for cell amount estimation on FCM imaging. When molecular analysis is required, the FCM evaluation may suggest the opportunity of an adjunctive needle pass to increase the cells availability. Behind the adequacy evaluation, we aimed to compare the microscopic assessment at FCM with the paired conventional cytological smears using the final histological diagnosis on surgical specimens as the gold standard. We demonstrated a substantial agreement between Vivascope sample classification and the FNA cytological report, although in some cases (4/20, 5%) the FCM classification was more accurate in the assessment of the risk of malignancy than the cytological diagnosis on smeared slides. In these cases, numbers 2 and 15 were recognized as malignant at FCM evaluation, while they were indeterminate and suspicious for malignancy, respectively, at conventional cytological examination; numbers 5 and 14 were suspicious for malignancy at FCM assessment, while they were reported as indeterminate high risk lesion (FN/SFN, TIR3B) on conventional cytological smears. The FCM advantage is probably due to the availability of confocal examination of the sample and to the specimen architecture preservation. The first allows a multiple layer assessment moving on the z axis along the fresh sample, offering a complete appraisal of the sampled material; the second is obtained by direct loading of cellular material on the matrix immediately after sampling, avoiding smearing or spinning alteration of the original tissue structure (Figure 2). When compared with final histology, one of the two Bethesda class III (AUS/FLUS) cases resulted in a malignant lesion, accordingly with recent studies demonstrating that patients with an FNA categorized as AUS/FLUS may have a higher risk of malignancy than traditionally believed [29,30]. The Italian TIR3A category has a lower risk of malignancy [31]; we have to take in account that we enrolled only thyroid nodules classified at medium-high risk of malignancy by ultrasound evaluation and addressed to surgery. FCM evaluation based on morphological characteristics such as architectural atypia, nuclear irregularities, and cytoplasmic features demonstrated high positive predictive value. All cases classified as suspicious for malignancy or malignant on digital images were confirmed to be cancers at final histology. Such positive predictive value is partially due to the preserved papillary structure and the evidence of irregular nuclear morphology and nuclear pseudoinclusions (Figure 1). No false positive or false negative results were registered in this study. Although the number of cases is limited, FCM seems a promising approach for thyroid cytology. Our results should be confirmed in a large cohort of patients. 

Of note, the FCM classification of thyroid cytology is available at the time of FNA sampling. Such bedside availability of diagnostic information may speed up patient management for appropriate treatment. This is not a crucial matter for most patients with thyroid nodules, but is a relevant issue to avoid both FNA repetition in pediatric age and diagnostic delay in critical patients with advanced disease. In the same way, FCM use may support cytological diagnostic of ultrasound suspicious cervical nodes by allowing a rapid appraisal of neoplastic cells, if present, and a consequent addressing of the patient to a tailored workup. Finally, instant digital images from FCM act as a whole slide scanning of digital pathology, since they may be shared for remote consulting, annotated for didactic purpose, or measured for prognostic or predictive factors. The learning curve for FCM images is rapid and similar to that required for conventional digital pathology [32]. Artificial Intelligence algorithms have been successfully developed directly on native digital FCM images for computer-aided diagnostics by automated detection of tumor ROI [33,34].

## 5. Conclusions

In conclusion, the use of FCMs in different areas of human pathology is rapidly expanding and our study describes a new application in the setting of thyroid FNA preoperative cytological evaluation. It highlights advantages in immediate adequacy assessment and diagnostic information, while preserving cellular specimens for permanent morphology and additional analysis, thus improving timely and accurate patient management.

## Figures and Tables

**Table 1:** Clinical features, Cytological classification, and Histological diagnosis. FA: follicular adenoma, PTC: papillary thyroid carcinoma, HB-PTC: hobnail subtype papillary thyroid carcinoma, FV-PTC Follicular variant papillary thyroid carcinoma, OXY-PTC Oncocytic subtype papillary thyroid carcinoma, TC-PTC tall cell subtype papillary thyroid carcinoma, HCC Oncocytic cell carcinoma (Hurthle cell carcinoma).

ID Patient	Sex	Age	Max Diameter (mm)	Clinical Features		FNA FCM	FNA		Histological Diagnosis
				TSH	US Eu-Tirads		ICCRTC	BSRTC	
**1**	F	61	7	0.6	5	Suspicous for malignancy	TIR5	Malignant	PTC
**2**	F	36	30	2.1	4	Malignant PTC	TIR3B	FN/SFN	PTC
**3**	M	49	50	0.8	3	Follicular lesion	TIR3A	AUS/FLUS	FA
**4**	F	30	19	1.6	5	Malignant PTC	TIR5	Malignant	PTC
**5**	F	48	7	1.5	4	Suspicous for malignancy	TIR3B	FN/SFN	PTC
**6**	F	51	18	1.2	5	Malignant PTC	TIR5	Malignant	PTC
**7**	M	65	16	3.2	5	Suspicous for malignancy	TIR4	SM	HB-PTC
**8**	F	72	10	0.56	4	Follicular lesion	TIR3A	AUS/FLUS	FV-PTC
**9**	F	60	19	1.6	3	Follicular lesion	TIR4	SM	FA
**10**	M	70	12	1,2	4	Suspicous for malignancy	TIR4	SM	PTC
**11**	F	49	10	1.4	4	Malignant PTC	TIR5	Malignant	PTC
**12**	F	36	8	1	4	Malignant PTC	TIR5	Malignant	PTC
**13**	F	47	9	1.3	5	Malignant PTC	TIR5	Malignant	PTC
**14**	F	72	14	1.7	4	Suspicous for malignancy	TIR3B	FN/SFN	FV-PTC
**15**	M	53	21	1.2	4	Malignant PTC	TIR4	SM	OXY-PTC
**16**	F	53	16	1	4	Follicular lesion	TIR3B	FN/SFN	HCC
**17**	M	82	70	2.1	5	Malignant PTC	TIR5	Malignant	TC-PTC
**18**	M	73	36	1	4	Follicular lesion	TIR3B	FN/SFN	HCC
**19**	F	35	12	2.1	3	Follicular lesion	TIR3B	FN/SFN	FA
**20**	F	53	23	1.1	3	Follicular lesion	TIR3B	FN/SFN	FA

**Table 2:** Molecular results investigated on 20 samples with Next Generation Sequency Technology. Legend: n.v. = not valuable; AF = Allele Frequency.

ID Patient	Cells/ Section	% Tumoral Cells	Mutational Analysis		RNA Fusion	
			Cytomatrix	Surgical Tissue	Cytomatrix	Surgical Tissue
**1**	3000	90%	BRAF	BRAF	-	-
			pVal600E/c.1799 T > A	pVal600E/c.1799 T > A		
			AF: 17.65%	AF: 43%		
**2**	1200	90%	BRAF	BRAF	-	-
			pVal600E/c.1799 T > A	pVal600E/c.1799 T > A		
			AF: 35.91%	AF: 36%		
**3**	1200	100%	Wildtype	Wildtype	Negative	Negative
**4**	2000	90%	Wildtype	Wildtype	Negative	Negative
**5**	600	70%	BRAF	BRAF	-	-
			pVal600E/c.1799 T > A	pVal600E/c.1799 T > A		
			AF: 20.95%	AF: 33.6%		
**6**	1500	70%	Wildtype	Wildtype	CUX1-RET.	CUX1-RET.
					C10R12	C10R12
**7**	1000	90%	BRAF	BRAF	-	-
			pVal600E/c.1799 T > A	pVal600E/c.1799 T > A		
			AF: 24.89%	AF: 10.84%		
**8**	250	80%	n.v.	Wildtype	n.v.	n.v.
**9**	700	90%	Wildtype	Wildtype	Negative	Negative
**10**	1000	70%	Wildtype	Wildtype	Negative	Negative
**11**	2200	80%	BRAF	BRAF	-	-
			pVal600E/c.1799 T > A	pVal600E/c.1799 T > A		
			AF: 38.11%	AF: 36.33%		
**12**	1800	80%	BRAF	BRAF	-	-
			pVal600E/c.1799 T > A	pVal600E/c.1799 T > A		
			AF: 36.11%	AF: 18.66%		
**13**	600	50%	Wildtype	Wildtype	Negative	Negative
**14**	700	80%	BRAF	BRAF	-	-
			pVal600E/c.1799 T > A	pVal600E/c.1799 T > A		
			AF: 41.68%	AF: 33.5%		
**15**	2500	70%	BRAF	BRAF	-	-
			pVal600E/c.1799 T > A	pVal600E/c.1799 T > A		
			AF: 20.99%	AF: 11.26%		
**16**	500	80%	n.v.	n.v.	n.v.	n.v.
**17**	1200	90%	Wildtype	Wildtype	Negative	Negative
**18**	1700	90%	HRAS	HRAS	-	-
			p.Gln61arg/c.182	p.Gln61arg/c.182		
			A > G	A > G		
			AF: 47.05%	AF: 46.11%		
**19**	3000	80%	Wildtype	Wildtype	Negative	Negative
**20**	600	80%	Wildtype	Wildtype	Negative	Negative

## Data Availability

The data presented in this study are available on request from the corresponding author. The data are not publicly available due to privacy or ethical restrictions.

## References

[1] Durante C., Hegedus L., Czarniecka A., Paschke R., Russ G., Schmitt F., Soares P., Solymosi T., Papini E. (2023). 2023 European Thyroid Association clinical practice guidelines for thyroid nodule management. Eur. Thyroid J..

[2] Tapoi D.A., Lambrescu I.M., Gheorghisan-Galateanu A.A. (2023). Preoperative evaluation of thyroid nodules–Diagnosis and management strategies. Pathol. Res. Pract..

[3] Capitanio A., Dina R.E., Treanor D. (2018). Digital cytology: A short review of technical and methodological approaches and applications. Cytopathology.

[4] Malvehy J., Pérez-Anker J., Toll A., Pigem R., Garcia A., Alos L.L., Puig S. (2020). Ex vivo confocal microscopy: Revolution in fast pathology in dermatology. Br. J. Dermatol..

[5] Longo C., Pampena R., Bombonato C., Gardini S., Piana S., Mirra M., Raucci M., Kyrgidis A., Pellacani G., Ragazzi M. (2019). Diagnostic accuracy of ex vivo fluorescence confocal microscopy in Mohs surgery of basal cell carcinomas: A prospective study on 753 margins. Br. J. Dermatol..

[6] Rocco B., Cimadamore A., Sarchi L., Bonetti L.R., Bertoni L., Azzoni P., Assumma S., Turri F., Bozzini G., Eissa A. (2021). Current and future perspectives of digital microscopy with fluorescence confocal microscope for prostate tissue interpretation: A narrative review. Transl. Androl. Urol..

[7] Sighinolfi M.C., Cimadamore A., Cassani A., Assumma S., Sarchi L., Filippi B., Turri F., Reggiani Bonetti L., Maiorana A., Eissa A. (2023). Digital real-time microscopy of ex-vivo tissues: A novel strategy to control surgical accuracy. Urologia.

[8] Krishnamurthy S., Sabir S., Ban K., Wu Y., Sheth R., Tam A., Meric-Bernstam F., Shaw K., Mills G., Bassett R. (2020). Comparison of Real-Time Fluorescence Confocal Digital Microscopy with Hematoxylin-Eosin-Stained Sections of Core-Needle Biopsy Specimens. JAMA Netw. Open.

[9] Titze U., Sievert K.D., Titze B., Schulz B., Schlieker H., Madarasz Z., Weise C., Hansen T. (2022). Ex Vivo Fluorescence Confocal Microscopy in Specimens of the Liver: A Proof-of-Concept Study. Cancers.

[10] Villarreal J.Z., Pérez-Anker J., Puig S., Xipell M., Espinosa G., Barnadas E., Larque A.B., Malvehy J., Cervera R., Pereira A. (2023). Ex vivo confocal microscopy detects basic patterns of acute and chronic lesions using fresh kidney samples. Clin. Kidney J..

[11] Krishnamurthy S., Ban K. (2022). Feasibility of using digital confocal microscopy for cytopathological examination in clinical practice. Mod. Pathol..

[12] Stigliano S., Crescenzi A., Taffon C., Covotta F., Hassan C., Antonelli G., Verri M., Biasutto D., Scarpa R.M., Di Matteo F.M. (2021). Role of fluorescence confocal microscopy for rapid evaluation of EUS fine-needle biopsy sampling in pancreatic solid lesions. Gastrointest. Endosc..

[13] Amendoeira I., Arcidiacono P.G., Barizzi J., Capitanio A., Cuatrecasas M., Di Matteo F.M., Doglioni C., Fukushima N., Fulciniti F., Ginès A. (2022). New digital confocal laser microscopy may boost real-time evaluation of endoscopic ultrasound-guided fine-needle biopsy (EUS-FNB) from solid pancreatic lesions: Data from an international multicenter study. EBioMedicine.

[14] Tessler F.N., Middleton W.D., Grant E.G., Hoang J.K., Berland L.L., Teefey S.A., Cronan J.J., Beland M.D., Desser T.S., Frates M.C. (2017). ACR Thyroid Imaging, Reporting and Data System (TI-RADS): White Paper of the ACR TI-RADS Committee. J. Am. Coll. Radiol..

[15] Haugen B.R., Alexander E.K., Bible K.C., Doherty G.M., Mandel S.J., Nikiforov Y.E., Pacini F., Randolph G.W., Sawka A.M., Schlumberger M. (2016). 2015 American Thyroid Association Management Guidelines for Adult Patients with Thyroid Nodules and Differentiated Thyroid Cancer: The American Thyroid Association Guidelines Task Force on Thyroid Nodules and Differentiated Thyroid Cancer. Thyroid.

[16] Nardi F., Basolo F., Crescenzi A., Fadda G., Frasoldati A., Orlandi F., Palombini L., Papini E., Zini M., Pontecorvi A. (2014). Italian consensus for the classification and reporting of thyroid cytology. J. Endocrinol. Investig..

[17] Cibas E.S., Ali S.Z. (2017). The 2017 Bethesda System for Reporting Thyroid Cytopathology. Thyroid.

[18] Lloyd R.V., Osamura R.Y., Klöppel G., Rosai J. (2017). Tumours of the thyroid gland. World Health Organization Classification of Tumours of Endocrine Organs.

[19] Puliatti S., Bertoni L., Pirola G.M., Azzoni P., Bevilacqua L., Eissa A., Elsherbiny A., Sighinolfi M.C., Chester J., Kaleci S. (2019). Ex vivo fluorescence confocal microscopy: The first application for real-time pathological examination of prostatic tissue. BJU Int..

[20] Mu C., Ming X., Tian Y., Liu Y., Yao M., Ni Y., Liu Y., Li Z. (2022). Mapping global epidemiology of thyroid nodules among general population: A systematic review and meta-analysis. Front. Oncol..

[21] Mulita F., Anjum F. (2023). Thyroid Adenoma. StatPearls [Internet].

[22] Stigliano S., Crescenzi A., Marocchi G., Taffon C., Verri M., Di Matteo F.M. (2023). A new tool for rapid evaluation of endoscopic ultrasound through the needle biopsy in pancreatic cystic neoplasm. Dig. Liver Dis..

[23] Kim N.E., Raghunathan R.S., Hughes E.G., Longstaff X.R., Tseng C.H., Li S., Cheung D.S., Gofnung Y.A., Famini P., Wu J.X. (2023). Bethesda III and IV Thyroid Nodules Managed Nonoperatively after Molecular Testing with Afirma GSC or Thyroseq v3. J. Clin. Endocrinol. Metab..

[24] Muri R., Trippel M., Borner U., Weidner S., Trepp R. (2022). The Impact of Rapid On-Site Evaluation on the Quality and Diagnostic Value of Thyroid Nodule Fine-Needle Aspirations. Thyroid.

[25] Kirbis I.S., Maxwell P., Fležar M.S., Miller K., Ibrahim M. (2011). External quality control for immunocytochemistry on cytology samples: A review of UK NEQAS ICC (cytology module) results. Cytopathology.

[26] Qin S.Y., Zhou Y., Li P., Jiang H.X. (2014). Diagnostic efficacy of cell block immunohistochemistry, smear cytology, and liquid-based cytology in endoscopic ultrasound-guided fine-needle aspiration of pancreatic lesions: A single-institution experience. PLoS ONE.

[27] Baloch Z.W., Asa S.L., Barletta J.A., Ghossein R.A., Juhlin C.C., Jung C.K., LiVolsi V.A., Papotti M.G., Sobrinho-Simões M., Tallini G. (2022). Overview of the 2022 WHO Classification of Thyroid Neoplasms. Endocr. Pathol..

[28] Nikiforova M.N., Mercurio S., Wald A.I., Barbi de Moura M., Callenberg K., Santana-Santos L., Gooding W.E., Yip L., Ferris R.L., Nikiforov Y.E. (2018). Analytical performance of the ThyroSeq v3 genomic classifier for cancer diagnosis in thyroid nodules. Cancer.

[29] Mulita F., Plachouri M.K., Liolis E., Vailas M., Panagopoulos K., Maroulis I. (2021). Patient outcomes following surgical management of thyroid nodules classified as Bethesda category III (AUS/FLUS). Endokrynol. Pol..

[30] Crescenzi A., Palermo A., Trimboli P. (2021). Cancer prevalence in the subcategories of the indeterminate class III (AUS/FLUS) of the Bethesda system for thyroid cytology: A meta-analysis. J. Endocrinol. Investig..

[31] Trimboli P., Ferrarazzo G., Cappelli C., Piccardo A., Castellana M., Barizzi J. (2022). Thyroid Nodules with Indeterminate FNAC According to the Italian Classification System: Prevalence, Rate of Operation, and Impact on Risk of Malignancy. An Updated Systematic Review and Meta-analysis. Endocr. Pathol..

[32] Bertoni L., Puliatti S., Reggiani Bonetti L., Maiorana A., Eissa A., Azzoni P., Bevilacqua L., Spandri V., Kaleci S., Zoeir A. (2020). Ex vivo fluorescence confocal microscopy: Prostatic and periprostatic tissues atlas and evaluation of the learning curve. Virchows Arch..

[33] Ruini C., Schlingmann S., Jonke Ž., Avci P., Padrón-Laso V., Neumeier F., Koveshazi I., Ikeliani I.U., Patzer K., Kunrad E. (2021). Machine Learning Based Prediction of Squamous Cell Carcinoma in Ex Vivo Confocal Laser Scanning Microscopy. Cancers.

[34] Davoli D., Verri M., Crescenzi A. Automated Diagnosis of Pancreatic Cancer through Deep Learning and Ex-vivo Fluorescence Confocal Laser Microscopy: A New Frontier in Digital Pathology. Proceedings of the ECDP2023.

